# Do we still need to talk about maternal morbidity and near miss?

**DOI:** 10.1590/0102-311XEN185223

**Published:** 2023-11-13

**Authors:** Sandra Costa Fonseca

**Affiliations:** 1 Instituto de Saúde Coletiva, Universidade Federal Fluminense, Niterói, Brasil.

In 2006, CSP published a systematic review on maternal near miss ^1^. International literature had already been discussing this event, but in Brazil, scientific production was still in its first steps. Now, in 2023, CSP revisits this topic in an article ^2^ and an exchange of letters to the editors ^3^ ^,^ ^4^.

The relevance of studying the entire continuum of events in the pregnancy-puerperium cycle and their different levels of severity - from uncomplicated pregnancies to maternal death - is unquestionable ^5^. In 2011, the World Health Organization (WHO) solidified concepts of maternal morbidity and proposed specific approaches ^6^. Other systematic reviews - national and international - have been published and knowledge has expanded ^7^ ^,^ ^8^ ^,^ ^9^. Journals in the field of obstetrics and public health have been the major sources of scientific production on the topic.

However, beyond the advances in research, how are health services monitoring severe maternal morbidity and maternal near miss?

The article by Ferreira et al. ^2^ brings a concrete proposal to make maternal near miss surveillance mandatory in Brazil. These authors suggest a model based on the Brazilian Information System for Notificable Diseases (SINAN) or the use of data from the Hospital Information System of the Brazilian National Unified Health System (SIH/SUS). Two letters to the editors in response to the article discuss the strengths and the weaknesses of the proposal and bring new contributions to monitoring, extending it to severe maternal morbidity ^3^ ^,^ ^4^. The adoption of a system similar to the Latin American Perinatal Information System (SIP; http://www.sipplus.org/), linked to the Latin American Center for Perinatology, Women and Reproductive Health (CLAP/SMR; https://www.paho.org/en/clap), is perhaps the most appropriate proposal for the Brazilian reality, considering the national expertise in information systems ^10^, the specificities of obstetric events, and the possibilities of joint analysis of data from countries in the region ^11^ ^,^ ^12^ ^,^ ^13^. Some Brazilian institutions have already established surveillance and research partnerships using SIP ^14^ ^,^ ^15^. The system could initially be implemented as a sentinel surveillance in other maternity hospitals across the country, until it is incorporated universally.

However, other questions become necessary in this debate. The first concerns the assignment of monitoring tasks. Maternal death surveillance, which is mandatory in Brazil and has been regulated since 2008, provided for hospital committees at municipal, state, and federal level ^16^. Are these committees responsible for the surveillance of severe maternal morbidity and maternal near miss? Currently, few studies address the work of the committees, but undoubtedly they have made important contributions, where they are properly implemented, improving information ^17^. But do we have enough committees? And will the committees be able to handle this additional task?

The second question is a consequence of the first. Within the composition of the committees and/or health units, which professionals will complete the surveillance forms necessary for any monitoring system? Are our health professionals well acquainted to the topic? Are the topics of severe maternal morbidity and maternal near miss covered in Medicine, Nursing, and Obstetrics courses?

Generally, the teaching of maternal mortality, information systems, and epidemiological surveillance is included in subjects in the area of public health. But this does not seem to be enough, given the many problems of underreporting and inadequate completion, even for the most serious event, which is maternal death ^18^ ^,^ ^19^ ^,^ ^20^.

Strengthening the committees quantitatively and qualitatively seems to be the answer, whatever the choice for monitoring severe maternal morbidity and maternal near miss. Including the topic in health curricula and training professionals in how to complete surveillance forms and death certificates can be another step towards qualifying the fight against severe maternal morbidity and maternal near miss.
